# Lysine acetylation regulates the subcellular localization and function of WRKY63

**DOI:** 10.1093/plphys/kiae492

**Published:** 2024-09-17

**Authors:** Yuan-Hsin Shih, Fu-Yu Hung, Pei-Yu Lin, Jian-Sheng Chen, Yi-Sheng Cheng, Songguang Yang, Keqiang Wu

**Affiliations:** Institute of Plant Biology, National Taiwan University, Taipei 10617, Taiwan; Institute of Plant Biology, National Taiwan University, Taipei 10617, Taiwan; Center for Sustainable Resource Science, RIKEN, Yokohama 230-0045, Japan; Institute of Plant Biology, National Taiwan University, Taipei 10617, Taiwan; Institute of Plant Biology, National Taiwan University, Taipei 10617, Taiwan; Institute of Plant Biology, National Taiwan University, Taipei 10617, Taiwan; Institute of Plant Biology, College of Life Science, National Taiwan University, Taipei 10617, Taiwan; Genome and Systems Biology Degree Program, College of Life Science, National Taiwan University, Taipei 10617, Taiwan; Guangdong Key Laboratory for New Technology Research of Vegetables, Vegetable Research Institute, Guangdong Academy of Agricultural Sciences, Guangzhou 510640, China; Institute of Plant Biology, National Taiwan University, Taipei 10617, Taiwan

Dear Editor,

Post-translational modifications (PTMs) modulate protein function in eukaryote cells. Protein acetylation is an evolutionarily conserved PTM affecting subcellular localization, stability, protein–protein interactions, DNA binding ability, or enzymatic activity in mammals (Friso and van Wijk 2015; Park et al. 2015). In contrast, the functions of protein acetylation in plants are poorly understood. The WRKY protein family is one of the largest groups of transcription factor families in plants (Van Aken et al. 2013; Li et al. 2016; Zheng et al. 2020). Recent studies indicated that the function and activity of WRKY proteins can be regulated by acetylation (Sarris et al. 2015; Zheng et al. 2020). The WRKY domain of the WRKY transcription factors including WRKY41, WRKY70, WRKY60, and WRKY33 can be acetylated, which abolishes their DNA binding abilities (Sarris et al. 2015). In addition, acetylation of WRKY53 is important for its transcription activity (Zheng et al. 2020). However, how acetylation of WRKYs affects their functions and activities remains elusive.

Our previous studies showed that WRKY63 plays an important role in vernalization-induced flowering in *Arabidopsis* (Hung et al. 2022). To investigate whether WRKY63 can be acetylated in *Arabidopsis*, we analyzed the immunoprecipitated WRKY63-GFP protein using mass spectrometry. One acetylation site, K31 of WRKY63, was detected in 3 independent replicate experiments, suggesting that K31 might be the major acetylation site of WRKY63 (Fig. 1, A and B). K31 is located within the WRKY63 activation domain (Kalde et al. 2003). The sequence alignment of the group-IIIa WRKY transcription factors showed that K31 is also conserved in WRKY64, but not in other group-IIIa WRKYs (Fig. 1, A and C).

**Figure 1.**
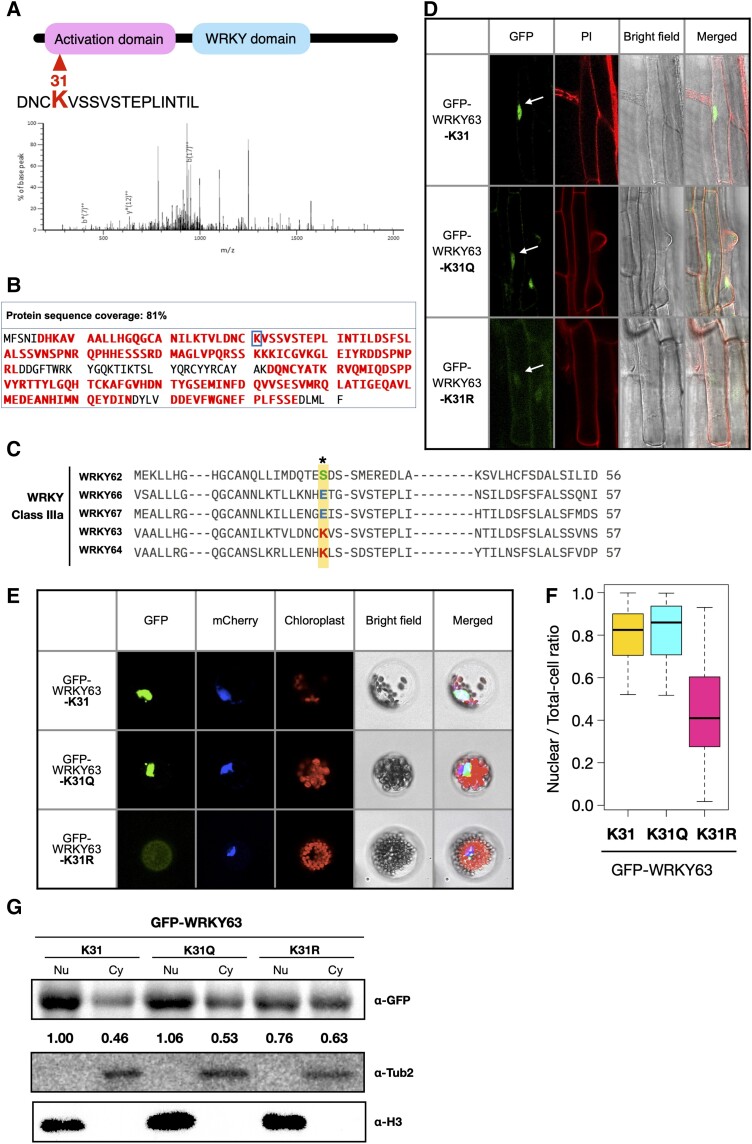
Acetylation affects the subcellular localization of WRKY63. **A)** Schematic representation of WRKY63 and the mass spectrometry spectrum of the WRKY63 peptide harboring acetylation at lysine 31. The activation domain and WRKY domain are marked. The horizontal axis represents mass-to-charge ratio (m/z). **B)** Acetylation of WRKY63 lysine identified from LC-MS/MS analysis. The GFP-WRKY63 protein was purified from 17-d-old 35S:GFP-WRKY63 transgenic plants using GFP magnetic agarose beads. After separation by SDS-PAGE, the 63 kDa band representing GFP-WRKY63 was selected for in-gel digestion using Asp-N endopeptidase cutting the N-terminal side of lysine, followed by LC-MS/MS analysis. LC-MS/MS analysis was performed in 3 replicates. The full length of WRKY63 was analyzed, with amino acids in red representing mapped sequences and the acetylated lysine 31 was marked by the blue box. **C)** Sequence alignment of the activation domains of the class IIIa WRKY proteins. The amino acid sequences predicted as activation domains contain about 50 amino acids (Kalde et al. 2003). K31 in WRKY63 is highlighted in red, and the aligned sites in other WRKY proteins are marked in blue or green. **D)** Subcellular localization of WRKY63 in transgenic plants. GFP fluorescence signals were observed in transgenic plants expressing wile type GFP-WRKY63-K31, GFP-WRKY63-K31Q, and GFP-WRKY63-K31R. The white arrow indicates the GFP signal in the nucleus. The cell wall is indicated by PI staining. **E)** GFP fluorescence signals of wild-type GFP-WRKY63-K31, GFP-WRKY63-K31Q, and GFP-WRKY63-K31R in *Arabidopsis* protoplasts. The nucleus is indicated by mCherry carrying a nuclear localization signal. The experiments were repeated 3 times with similar results. **F)** The ratio of the nuclear/total cell GFP fluorescence. GFP fluorescence was measured in 90 to 100 cells for each construct. The experiment was repeated 3 times with similar results. **G)** Western blot of GFP-WRKY63-K31, GFP-WRKY63-K31Q, and GFP-WRKY63-K31R. The nuclear (Nu) and cytosolic (Cy) proteins were extracted from GFP-WRKY63-K31, GFP-WRKY63-K31Q, and GFP-WRKY63-K31R transgenic plants. α-Tubulin (Tub2) and histone H3 were used as cytosolic and nuclear marks, respectively. The GFP signals were quantified by Image J.

To investigate whether K31 acetylation affects the WRKY63 function, we generated transgenic plants expressing wild-type WRKY63 (*35S:GFP-WRKY63-K31*), glutamine substitution mutation for acetylation-mimic WRKY63 (*35S:GFP-WRKY63-K31Q*), and arginine substitution mutation for non-acetylation-mimic WRKY63 (*35S:GFP-WRKY63-K31*R) in the *wrky63* mutant (*abo3*) background. We analyzed the expression of transgenes in the transgenic plants by RT-qPCR and western blot analysis. The transcript and protein levels of the transgenes are similar in *35S:GFP-WRKY63-K31*, *35S:GFP-WRKY63-K31Q*, and *35S:GFP-WRKY63-K31*R transgenic plants (Supplementary Fig. S1, A and B), suggesting that K31 acetylation does not affect the protein stability of WRKY63. We further analyzed WRKY63-GFP lysine acetylation levels by immunoblotting using an anti-acetylated lysine antibody. Similar levels of WRKY63-GFP lysine acetylation were observed in in *35S:GFP-WRKY63-K31*, *35S:GFP-WRKY63-K31Q*, and *35S:GFP-WRKY63-K31*R transgenic plants (Supplementary Fig. S2), suggesting that in addition to K31, other lysine residues may also be acetylated. WRKY63 was reported to be involved in ABA responses in *Arabidopsis* (Ren et al. 2010). Similar acetylation levels of WRKY63 were observed in *35S:GFP-WRKY63*, *35S:GFP-WRKY63-K31Q*, and *35S:GFP-WRKY63-K31R* plants with or without ABA treatment, indicating that ABA does not alter the acetylation level of WRKY63 (Supplementary Fig. S2). To analyze whether acetylation of WRKY63 affects the protein structure, we used Alphafold to analyze the structure change. The protein structure comparison of WRKY63-K31, WRKY63-K31Q, and WRKY63-K31R showed that K31 acetylation may not cause significance structure changes on WRKY63 (Supplementary Fig. S3).

We found that the GFP signal of GFP-WRKY63-K31 and GFP-WRKY63-K31Q was mainly located in the nucleus, whereas GFP-WRKY63-K31R showed decreased nuclear localization but increased cytosolic localization in the transgenic plants (Fig. 1D). Similar results were also observed when GFP:WRKY63, GFP:WRKY63-K31Q, or GFP:WRKY63-K31R was expressed in *Arabidopsis* protoplasts (Fig. 1E). To quantify the distribution of the GFP signal in the nucleus and cytosol, we calculated the ratio of nuclear fluorescence to total cell fluorescence. The nuclear/total cell fluorescence ratio of GFP-WRKY63 and GFP-WRKY63-K31Q was over 80%. By contrast, the nuclear/total cell fluorescence ratio of GFP:WRKY63-K31R was decreased to around 40% (Fig. 1F). The distribution of WRKY63 in the nucleus and cytosol was further analyzed by western blot using nuclear and cytosolic proteins extracted from GFP-WRKY63-K31, GFP-WRKY63-K31Q, and GFP-WRKY63-K31R transgenic plants. The results showed that the GFP-WRKY63-K31 and GFP-WRKY63-K31Q proteins were mainly localized in the nucleus, whereas GFP-WRKY63-K31R was equally distributed in the nucleus and cytosol (Fig. 1G). These results indicate that K31 acetylation regulates the cellular localization of WRKY63.

We previously reported that *wrky63* (*abo3*) mutant plants displayed an early flowering phenotype (Hung et al. 2022). The early flowering phenotype of the *wrky63* mutant (*abo3*) was rescued in plants transformed with wild-type GFP-WRKY63-K31 or GFP-WRKY63-K31Q, but not with GFP-WRKY63-K31R, indicating that K31 acetylation is crucial for WRKY63 function in regulating flowering time (Fig. 2, A and B). WRKY63 was reported to be involved in flowering by regulating the expression of *FLC* (Hung et al. 2022). Similar expression levels of *FLC* were observed in the wild-type Col-0, *35S:GFP-WRKY63-K31*, and *35S:GFP-WRKY63-K31Q*, but not in *35S:GFP-WRKY63-K31R* (Fig. 2C). These results suggest that the acetylation status of WRKY63 at K31 affects the flowering time through modulating *FLC* expression.

**Figure 2.**
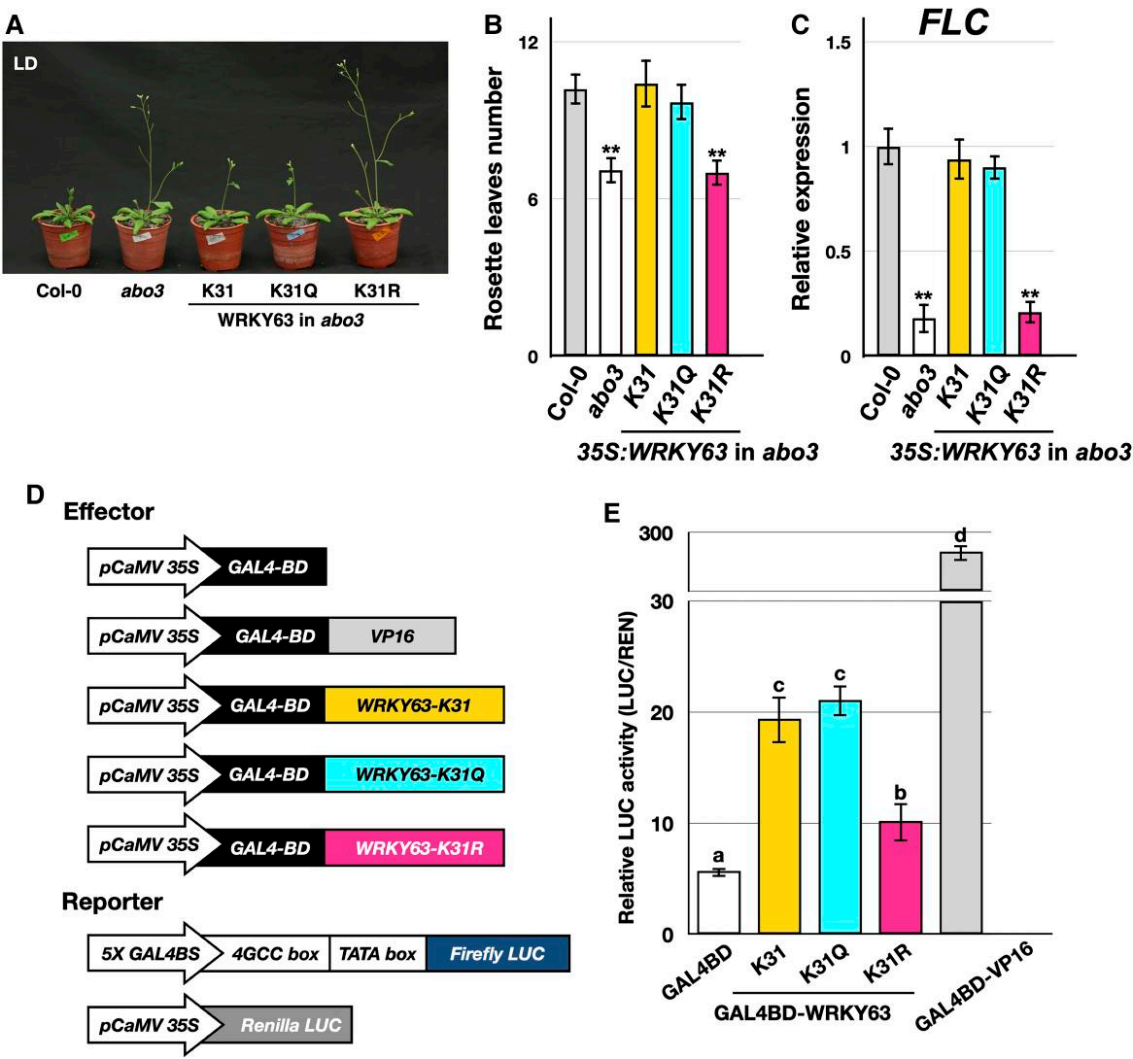
Acetylation affects the function of WRKY63. **A to C)** Flowering phenotypes **A)**, rosette leaf numbers at bolting **B)**, and *FLC* expression **C)** of Col-0, *wrky63* (*abo3*), and transgenic plants expressing wild-type GFP-WRKY63-K31, GFP-WRKY63-K31Q, or GFP-WRKY63-K31R in the *wrky63* (*abo3*) mutant background. Data are shown in means ± SD. **P < 0.05 (Student's *t*-test). At least 3 independent biological replicates were performed with similar results. **D)** Constructs used for transient transcriptional activity assays. **E)** Relative LUC activity from transient transcriptional activity assays. The relative LUC activity was calculated as firefly LUCIFERASE (LUC)/renilla LUCIFERASE (REN). Values are presented as means ± SD. Different lowercase letters present significant differences in data with 1-way ANOVA with LSD (*P* < 0.05). The experiments were repeated 3 times with similar results.

WRKY63 has been identified as a transcription activator (Ren et al. 2010; Hung et al. 2022). To investigate whether K31 acetylation affects the transcriptional activation ability of WRKY63, we conducted transient transcriptional activity assays using *Arabidopsis* protoplasts. The yeast GAL4 DNA binding domain fused with WRKY63-K31, WRKY63-K31Q, or WRKY63-K31R was analyzed for activating the firefly LUCIFERASE (LUC) reporter driven by a promoter containing the GAL4 binding sites (GAL4BS). The GAL4 DNA binding domain fused with the activation domain of VP16 (GAL4BD-VP16) was used as a positive control (Fig. 2D). GAL4BD-WRKY63-K31 and GAL4BD-WRKY63-K31Q showed similar transactivation activities, whereas GAL4BD-WRKY63-K31R had reduced transactivation activities (Fig. 2E). These results suggest that K31 acetylation is important for the transcriptional activity of WRKY63.

In conclusion, our study revealed a key lysine acetylation site affecting WRKY63 subcellular localization and activity. Although the mechanism how WRKY63 acetylation is regulated remains unknown, our findings uncover a novel regulatory mechanism of regulating the function of WRKYs through acetylation.

## Supplementary Material

kiae492_Supplementary_Data

## Data Availability

The data underlying this article are available in the article and in its online supplementary materials.

## Supplementary data

The following materials are available in the online version of this article.

**Supplementary Figure S1.** The expression of WRKY63-GFP in transgenic plants.

**Supplementary Figure S2.** The effect of ABA on WRKY63 acetylation.

**Supplementary Figure S3.** Protein structure comparison of WRKY63-K31, WRKY63-K31Q, and WRKY63-K31R.

**Supplementary Table S1.** Primers used in this study.
