# Supplementary figures and images for: Lysine acetylation regulates the subcellular localization and function of WRKY63
Source: Plant Physiol. 2024 Sep 17;196(4):2279–82. doi: 10.1093/plphys/kiae492 (PMC11637992; doi:10.1093/plphys/kiae492)

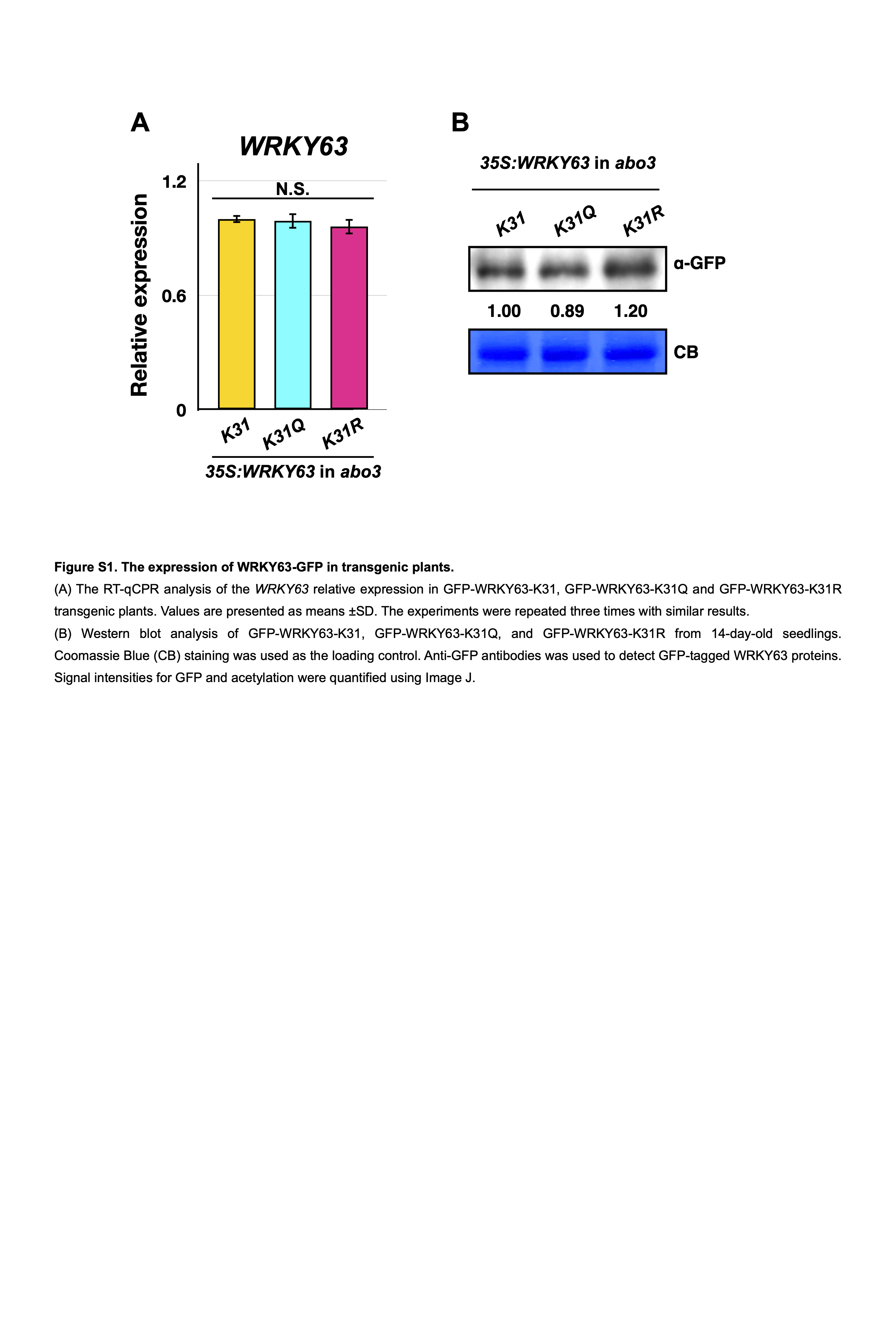

Supplement: kiae492_Supplementary_Data [file kiae492_supplementary_data.zip › WRKY63_edit_FigureS1.tiff]

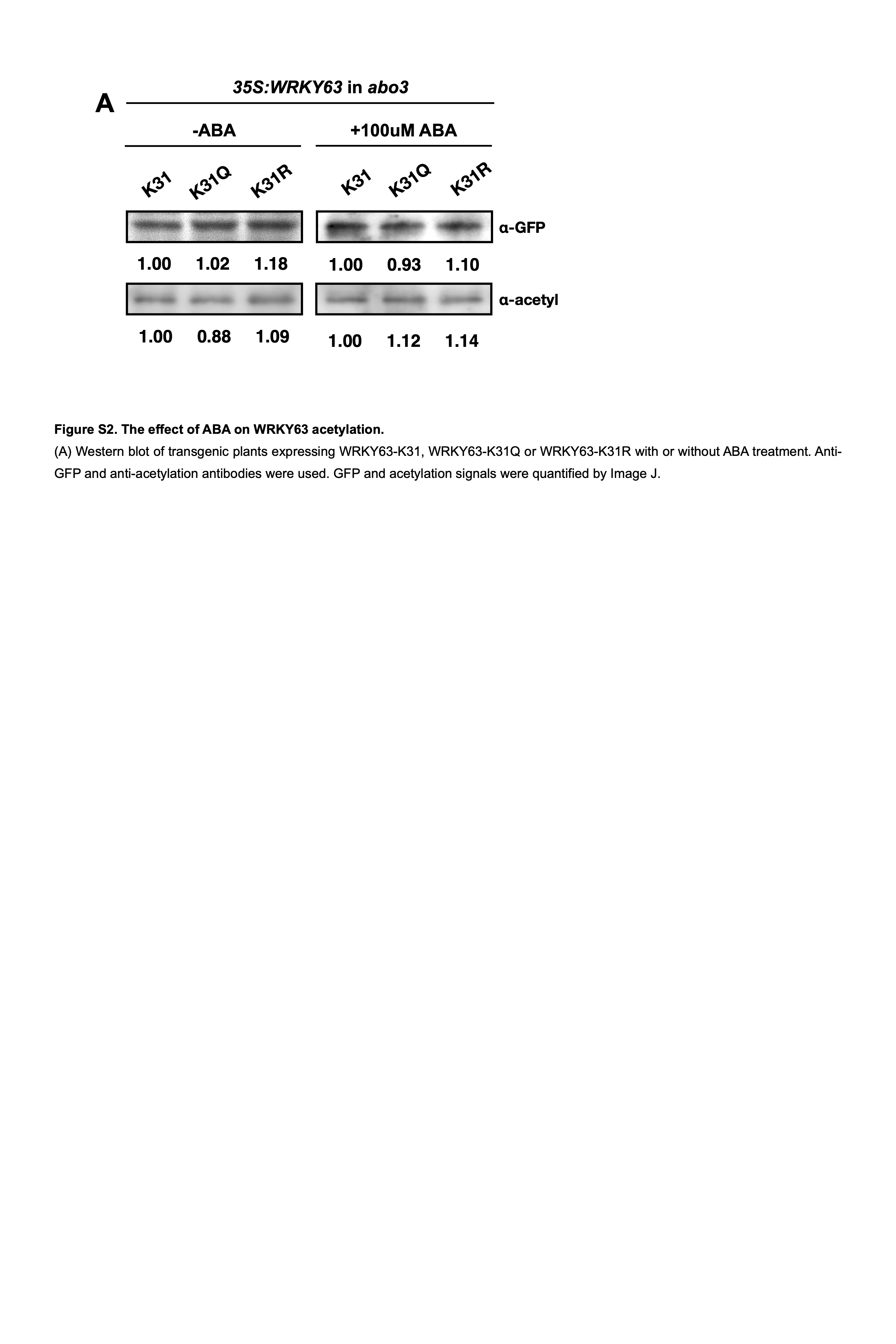

Supplement: kiae492_Supplementary_Data [file kiae492_supplementary_data.zip › WRKY63_edit_FigureS2.tiff]

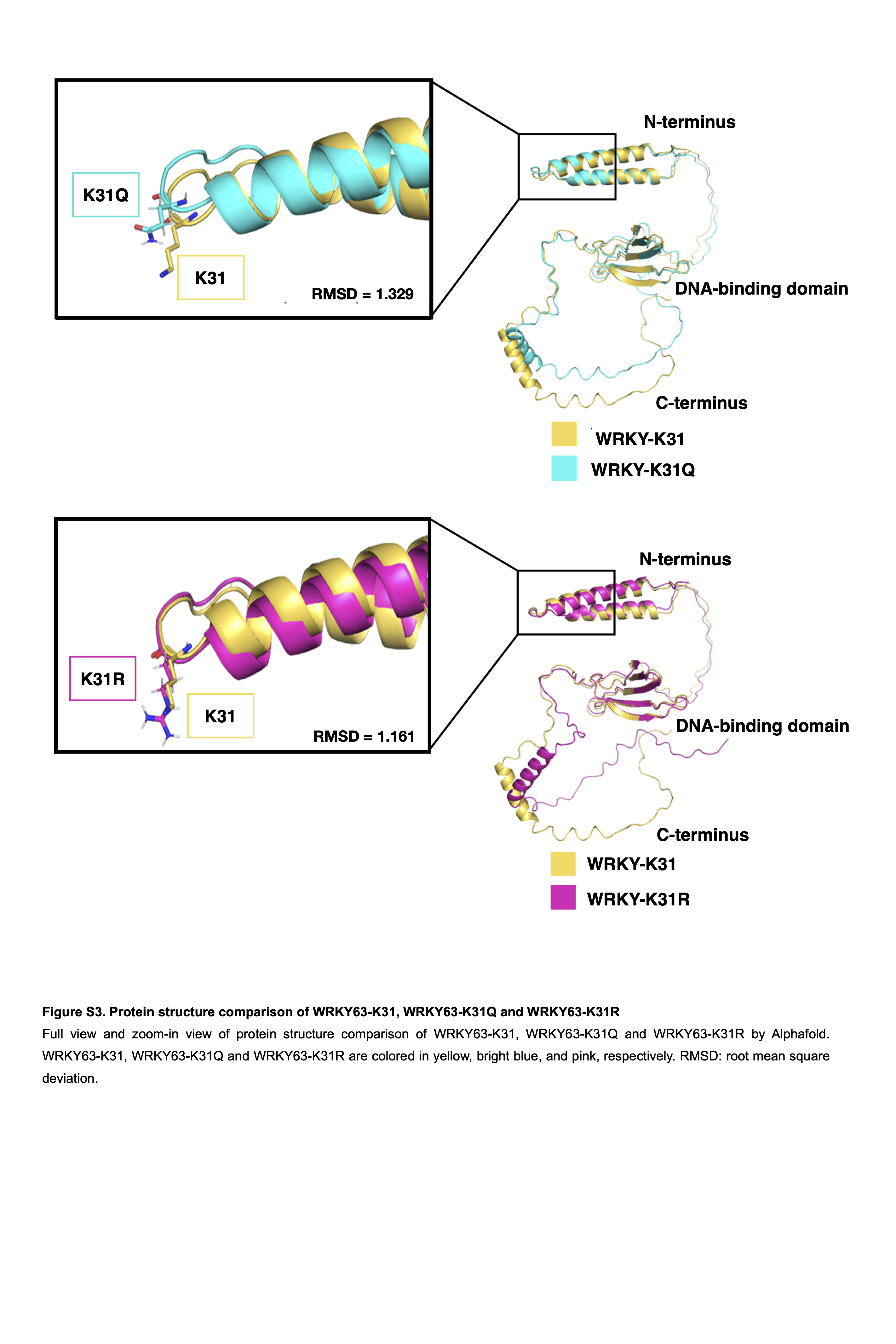

Supplement: kiae492_Supplementary_Data [file kiae492_supplementary_data.zip › WRKY63_edit_FigureS3.tiff]
